# A case of gastric heterotopic pancreas with gastroduodenal invagination

**DOI:** 10.1186/s40792-019-0669-7

**Published:** 2019-07-10

**Authors:** Shoko Iwahashi, Masaaki Nishi, Toshiaki Yoshimoto, Hideya Kashihara, Chie Takasu, Takuya Tokunaga, Tomohiko Miyatani, Jun Higashijima, Kozo Yoshikawa, Yuma Wada, Yoshimi Bando, Mitsuo Shimada

**Affiliations:** 1 Department of Surgery, Tokushima University, Tokushima, 770-8503 Japan; 2 Department of Division of Pathology, Tokushima University, 3-18-15 Kuramoto-cho, Tokushima, 770-8503 Japan

**Keywords:** Gastric heterotopic pancreas, Gastroduodenal invagination

## Abstract

**Background:**

Heterotopic pancreas (HP) is a rare disease commonly found incidentally on imaging studies, at endoscopy or at autopsy and can be associated with abdominal pain, vomiting, heart burn, gastric outlet obstruction, and even dysphagia in very rare cases. Heinrich’s classified HP into three groups, types1–3, with Heinrich’s type 3 HP the rarest and difficult to diagnose properly because it has only pancreatic ducts but has no islet and acini. The aim of this study is to report a case of gastric outlet obstruction caused by type 3 HP with gastroduodenal invagination with reference to the literature and diagnosed finally by immuno-histochemical analysis.

**Case presentation:**

The case presented is a 40-year-old male presenting with vomiting and abdominal pain. Computed tomography (CT) revealed a cystic mass in the upper abdomen and he was referred to the Tokushima University. Gastric fiber showed that the pedunculated mass originated from the stomach. An open distal gastrectomy was performed. Pathologically, there was small glands proliferation in the sub-mucosal (SM) layer which was membrane and cytoplasm (MUC)1 positive and muscle proliferation.

**Results:**

This finding revealed the tumor as HP. Postoperative course was uneventful and the patient was discharged 12 days after surgery. The patient has remained well 12 months after surgery.

**Conclusions:**

HP should be considered in the differential diagnosis of SM tumors with gastroduodenal invagination even if this is a rare symptom.

## Background

Submucosal (SM) tumors are tumors in the submucosa or muscularis propria of the gastric wall and are associated with many types of diseases. Gastrointestinal stromal tumors (GISTs) and leiomyomas are the most common gastric SM tumors. Heterotopic pancreas (HP) is also one of the differential diagnoses for SM tumors but is very rare. It is often difficult to differentiate SM tumors [1, 2], so some imaging examination, such as endoscopic ultrasound-guided fine-needle aspiration (EUS-FNA) which has been recently reported to be useful [3] is essential for a precise diagnosis.

As previously stated, HP is one of the SM tumors and is defined as the presence of pancreatic tissue outside its normal location and without anatomic and vascular continuity with the main body of the pancreas [1]. Usually asymptomatic, gastric heterotopia is commonly an incidental finding on imaging studies, at endoscopy or at autopsy [1, 4]. Only a few cases are shown to be clinically significant, particularly larger lesions, which can be associated with abdominal pain, vomiting, gastric outlet obstruction, and even dysphagia in the very rare cases located near the esophago-gastric junction [5]. Gastric HP is commonly situated in the submucosa of the distal stomach, most often within 50 mm of the pylorus. It is usually seen at endoscopy as a yellowish, soft tumor. The SM location of HP is often evaluated by endoscopic ultrasound with the recognition of features distinguishing it from GISTs and leiomyoma, the major clinical and endoscopic differential diagnoses [4].

Pathologically, HP is variably composed of pancreatic acini, ducts, or islets. Some cases contain all components of the pancreas, including ducts, acini, and endocrine islets, whereas others consist of pancreatic ducts only. Heinrich named the three types as types 1–3 respectively [6]. HPs have also been termed ‘adenomyomas’ or ‘myoepithelial hamartomas’ [1, 7] and are usually characterized by dilated pancreatic ducts only and surrounded by prominent smooth muscle proliferation. Other cases consist of only acinar tissue, whereas some consist of only islet cells. This study reports a case of gastric outlet obstruction with gastroduodenal invagination of HP with reference to the literature.

## Case presentation

**Fig. 1:**
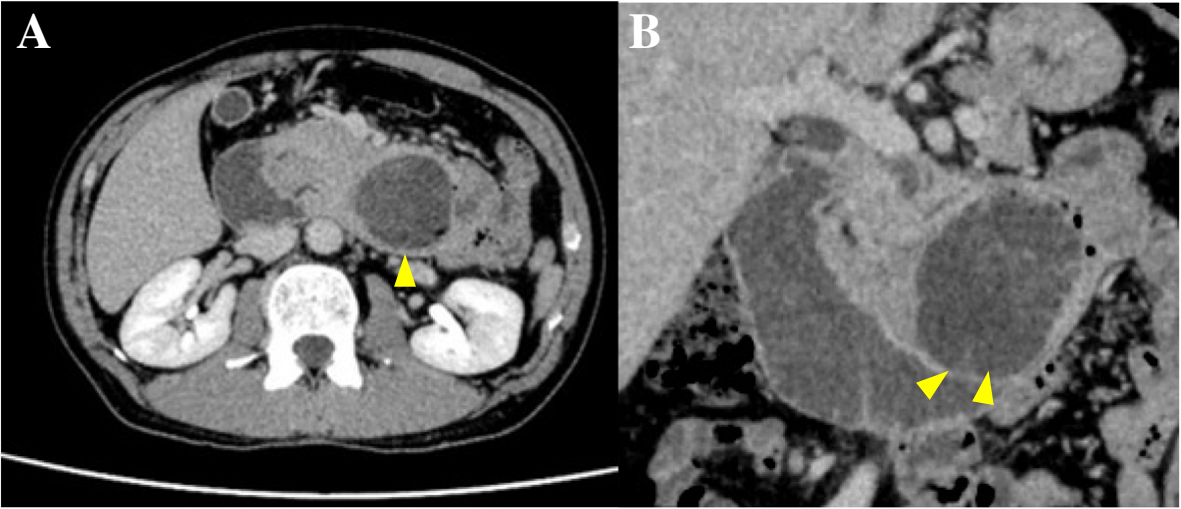
Abdominal CT showed 6 cm low-density mass in the retroperitoneum, in all phase (arrow). **a** Coronal view showed gastroduodenal invagination. **b** Duodenum proximal to the mass is dilatated (arrow)

**Fig. 2:**
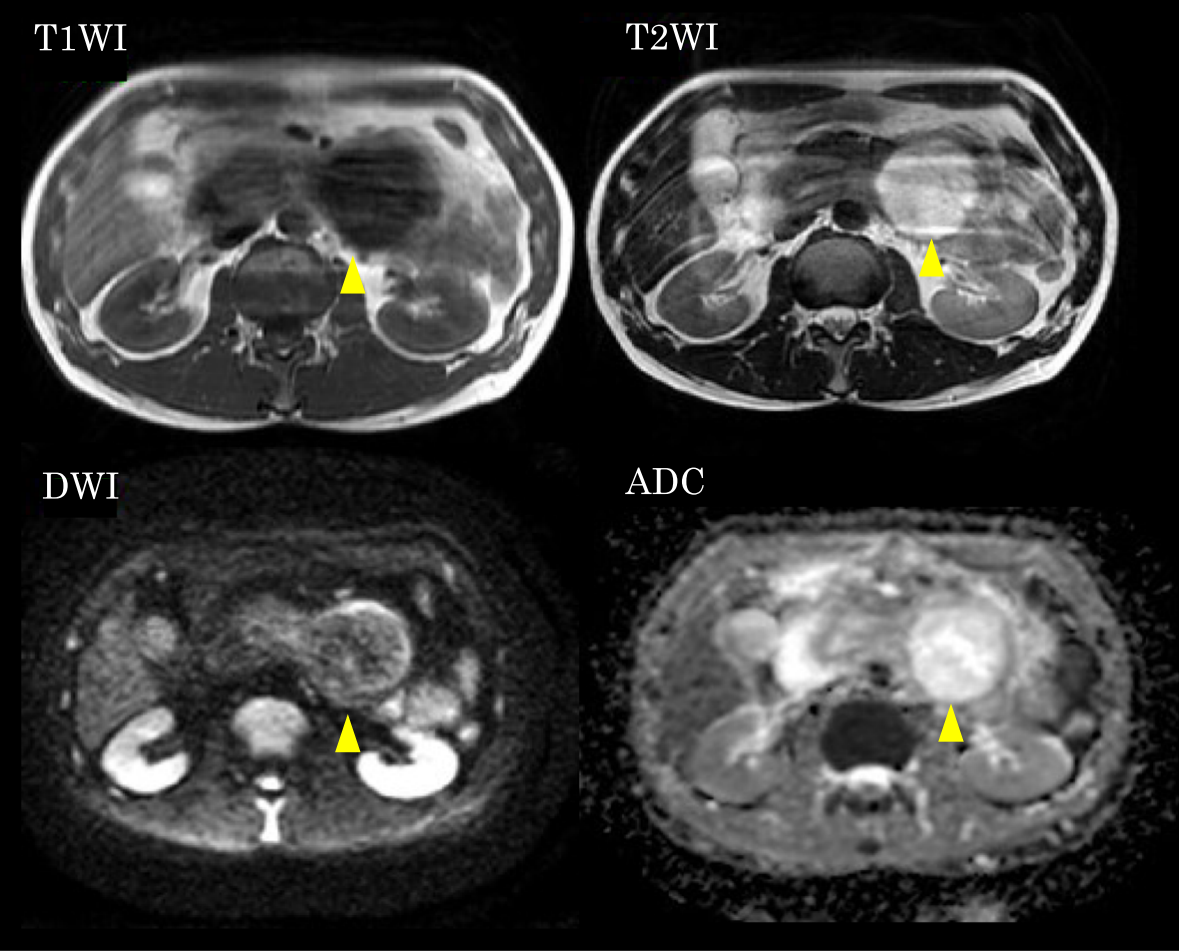
MRI showed T1 WI low, T2 WI high, ADC high, DWI high mass (arrow)

**Fig. 3:**
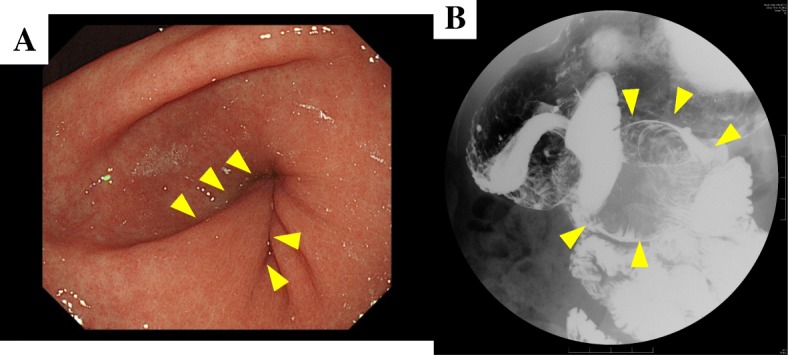
Gastric fiber showed the pedunculated mass was originated from the stomach, which invaginated into the duodenum (arrow) (**a**). Gastric fluoroscopy showed the mass invaginated to the duodenum (**b**)

A 40-year-old male presented with vomiting and abdominal pain. Computed tomography (CT) revealed a cystic mass in the upper abdomen and the patient was subsequently referred to the Tokushima University. Laboratory tests were as follows: leukocyte count 7400/μl; hemoglobin 8.6 g/dl; albumin 4.0 g/dl; amylase 91 IU/l; total bilirubin 0.3 mg/dl; carcinoembryonic antigen (CEA) 1.5 ng/ml; carbohydrate antigen 19-9 (CA19-9) 8 U/mL; DUPAN-II < 25 U/ml; and S-pancreas-1 antigen (Span-1) 7 U/ml. The CT in the axial view revealed a 6 cm low-density mass in the pylorus of the stomach and a coronal view confirmed gastroduodenal invagination (Fig. 1a, b). Magnetic resonance imaging (MRI) revealed a mass lesion: T1-weighted image (WI) low, T2 WI high, intermediate-high apparent diffusion coefficient (ADC) value, and diffusion-weighted image (DWI) high (Fig. 2). The gastric fiber showed that the pedunculated mass originated from the stomach and the cushion sign was positive (Fig. 3). The tumor was expected to be difficult to resect by endoscopy because of its size and the presence of gastroduodenal invagination. We did not perform ultrasonography (US).

**Fig. 4:**
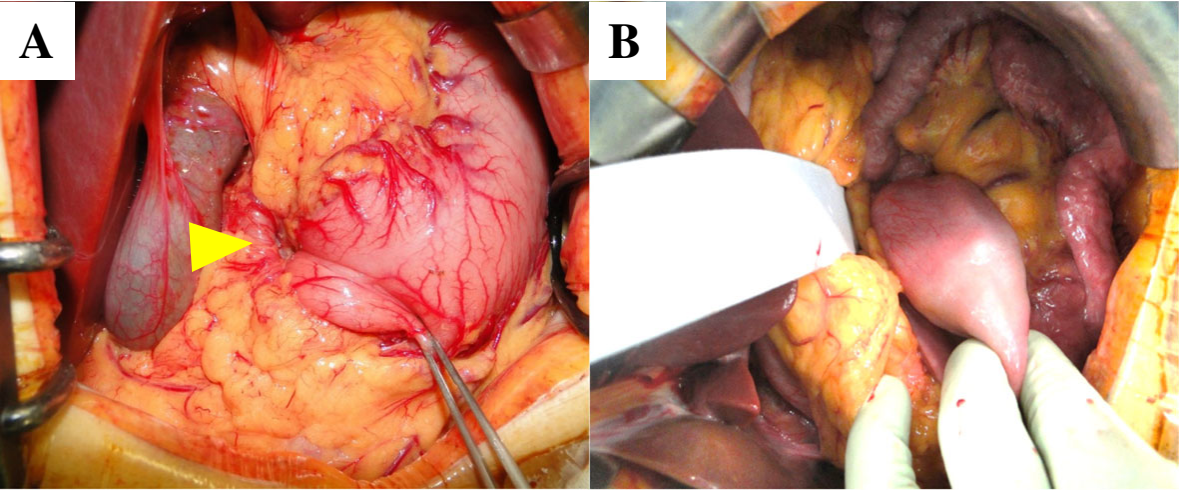
Intraoperative finding. Open distal gastrectomy was performed. The mass was found in the duodenum 3rd portion and invaginated to the duodenum (arrow) (**a**). We moved it to the stomach and performed distal gastrectomy with B-1 reconstruction (**b**)

According to these findings, GIST, duplication cyst, malignant lymphoma, and gastric polyp could be stated as a differential diagnosis (Table 1). In particular, MRI finding suggested duplication cyst but gastric duplication has not reported to be accompanied by gastroduodenum invagination. Therefore, the preoperative diagnosis was a SM cystic tumor originating from the stomach with gastroduodenal invagination. An open distal gastrectomy was performed because of its invagination at which time the mass was found in the duodenum 3rd portion and invaginated to the duodenum. It was moved to the stomach and a distal gastrectomy with B-1 reconstruction was performed (Fig. 4). It was unclear whether there was malignant finding.

**Table 1:** Preoperative different diagnosis and typical findings by MRI, CT, and US

	This case	GIST	Gastric duplication	Malignant lymphoma	Gastric polyp
CT	Hypo-vascular	Hyper	Hypo	Iso	Hypo
US	No data	Iso-low	Low	Low	Iso-low
MRI					
T1	Low	Low	Low	Iso	Not high
T2	High	High	High	Intermediate	Not high
DWI	Not high	High	Not high	High	Not high

**Fig. 5:**
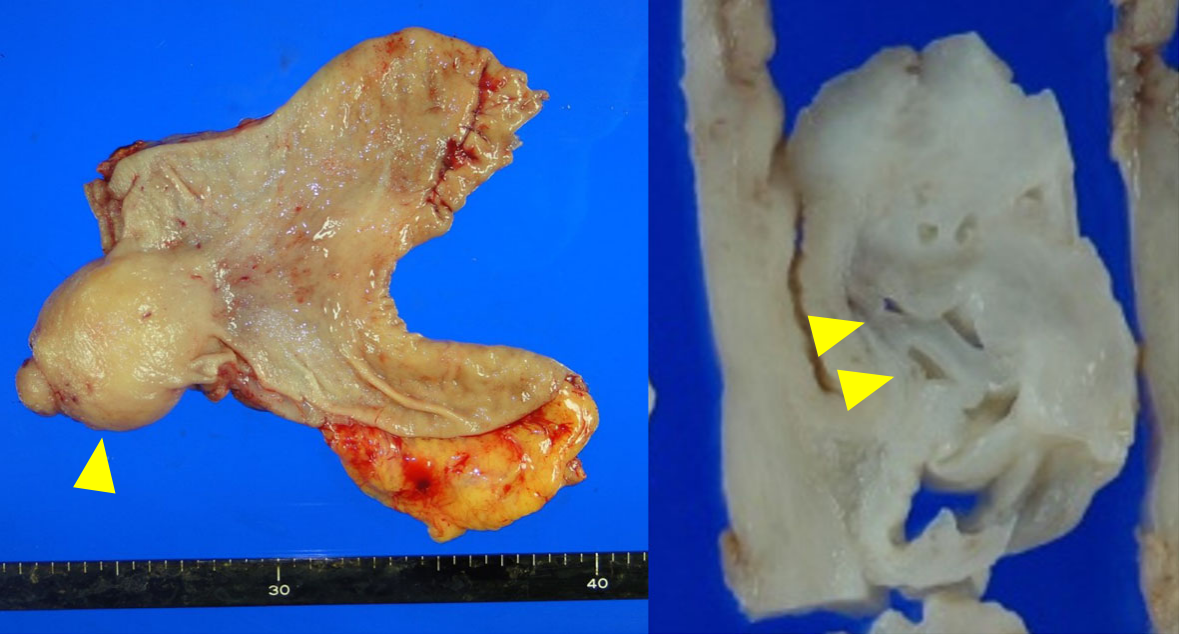
Resected specimen showed solid nodule originated from gastric antrum

**Fig. 6:**
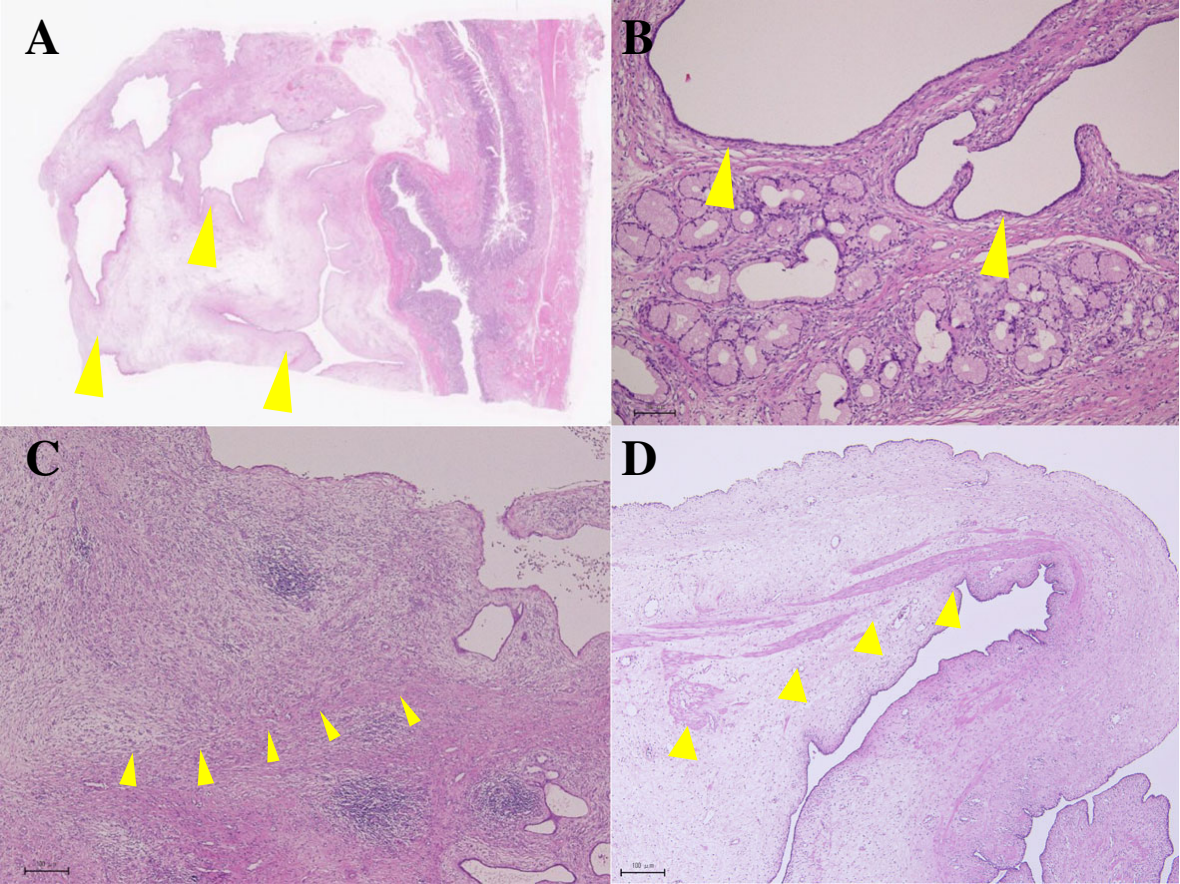
Heterotopic glands (arrow) and stroma was found in the SM layer (**a**) and delated grands (arrow) was found (**b**). Edematous stroma and inflammatory cells were found in SM layer (**c**). Smooth muscle bundles were found around the grands (arrow) (**d**)

**Fig. 7:**
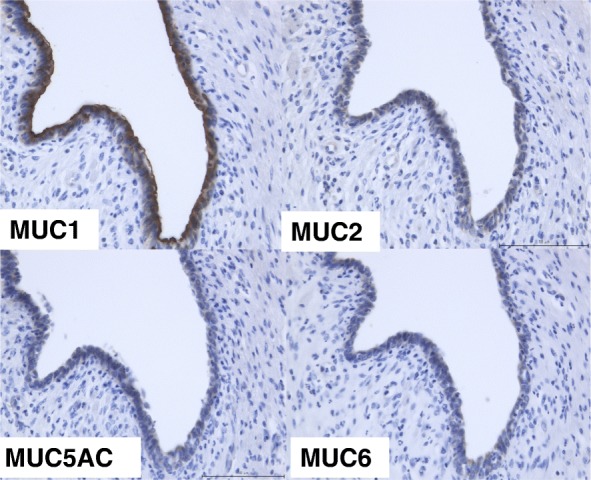
Immunohistochemistry, this tumor is MUC1(+), MUC2(−), MUC5AC(−), MUC6(−)

The resected specimen showed a solid, edematous nodule with small glands originating from the gastric antrum with some of the small glands and the stroma in the SM layer. (Fig. 5). Pathologically, many glands and stroma were found in the SM layer some of these glands were dilated (Fig. 6a, b). Edematous stroma and inflammatory cells were found in SM layer and smooth muscle bundles were found around the glands (Fig. 6c, d). At first, it was diagnosed as SM heterotopic glands of the stomach. However, it was difficult to differentiate from other SM cysts. Immuno-histochemical analysis was performed, which showed positivity on the grands’ membranes and cytoplasm (MUC)1 and negative on MUC2, MUC5AC, and MUC6, which suggested that the grands originated from pancreatic ducts (Fig. 7). This tumor lacked pancreatic acini but the above results of immuno-staining revealed that these atypical tissues were pancreatic ducts and the tumor was HP. The postoperative course was uneventful and the patient was discharged 12 days after surgery and has remained well 12 months after surgery.

## Discussion

SM tumors are divided into a number of groups and GISTs and leiomyomas are the most common of these gastric SM tumors. HP is also one of the differential diagnoses. It is often difficult to differentiate SM tumors into the respective groups [5]. Pang’s retrospective study of thirty-two cases of HP showed that there were no cases which were diagnosed properly before the operations were performed [8]. Regarding an endoscopic study, Kubota’s review of 26 cases of SM tumor suggested that most HPs are yellowish, cloudy, small, soft, and nodular in shape [4]. On the one hand, solid tumors such as neuro-endocrine tumors (NETs) generally extend vertically and are papillary while; on the other hand, soft tumors such as HPs tend to extend horizontally and show cushion sign positive. The case described in this study showed a cushion sign positive which means a soft tumor but with a formed papillary architecture and gastroduodenal invagination, atypical for a soft tumor like HP. And the correlation pedunculated shape and HP’s pathological type was obscure.

The SM location of pancreatic heterotopia is often evaluated using endoscopic ultrasound with the recognition of features distinguishing it from a GIST or leiomyoma, the major clinical and endoscopic differential diagnoses. Moreover, EUS-FNA is reported to be useful [3] for a precise diagnosis. In the case study, endoscopic biopsy could have been performed at the same time but tissue specimens obtained using standard endoscopic biopsy forceps would not have been adequate for histopathological diagnosis of HP [9].

It has been reported that the proper diagnosis rate of HP by CT is only 16.7% [10], while the proper diagnosis rate using MRI is higher. MRI often shows HP as T1 WI high, T2 WI low [11]. In the case study, the mass lesion showed low intensity in T1 WI and high intensity in T2 WI. The density of the tumor’s content suggested that this tumor included water-like content and resembled that of a pancreatic serous cystic neoplasm (SCN) and gastric duplication and was atypical as HP or a GIST. However, the resected specimen was filled with solid tumor. Pathologically, there were many secretory glands and this stroma was edematous, which might show a T2 WI, water-like image. The correlation between image by CT and MRI and pathological type of HP is obscure; however, some reports suggested that the enhancement on CT is typically similar to that of the normal pancreas but the enhancement degree depends heavily on the histopathologic composition. Duct dominant type including type 3 HP exhibits lower enhancement than the normal pancreas [12]. MRI could show the variable image according to HP’s subtype.

Pathologically, HP is variably composed of pancreatic acini, ducts, or endocrine islets. Some cases contain all components of the pancreas, including ducts, acini, and endocrine islets, whereas others consist of pancreatic ducts only. In 1909, Heinrich classified the HP into three types histologically: type 1, the most common, with all the components of the pancreas including acini, ducts, and endocrine islets; type 2, with acini and ducts and no endocrine islets; and type 3, with ducts alone [6]. In the case study, immuno-histochemical analysis showed MUC1-positive cells, which revealed the existence of benign pancreatic ducts, identifying it as belonging to type 3. They are usually characterized by only dilated pancreatic ducts and are surrounded by prominent smooth muscle proliferation while some of them are cystically dilated [7]. In type 3, because only the pancreatic ducts exist, differentiation between HP and SM heterotopic glands is very difficult. The case study was diagnosed as HP because of two reasons. First, the mass expressed MUC1 on the glands’ membranes, which was expressed specifically in the pancreatic glands’ membranes and cytoplasm. Secondly, the muscle proliferation existed in the mass which was also specific to type 3 HP.

HP is usually asymptomatic [1, 13, 14] and commonly found incidentally on imaging studies, at endoscopy or at autopsy. Only a few cases are clinically significant, particularly larger lesions, because they can be associated with abdominal pain, vomiting [15] and gastric outlet obstruction [5, 16]. Dysphagia and heartburn can occur in the very rare cases when the lesion is located near the esophago-gastric junction [2, 17, 18]. Gastroduodenal invagination is a rare symptom and to the best of the authors’ knowledge, only a few cases of HP as a cause of gastric outlet obstruction in adult have been observed [5, 15, 16, 19–23]. Almost all gastric HP is situated in the submucosa of the distal stomach, and often within 50 mm of the pylorus [1]. This location and the larger size of a tumor may lead to several symptoms similar to the case study where the lesion was near the pylorus and 6 cm in size [1]. About treatment of HP, if HP is discovered as an incidental finding, local excision is recommended. Increasingly, some are removed endoscopically with satisfactory postoperative results [16]. However, endoscopic excision can be considered in select case. In our case, the reduction of incarceration was expected to be difficult endoscopically and laparoscopically, and open strategy was performed. Moreover, the adenocarcinoma could not be excluded completely and DG was performed. In some case, DG was also performed because of obscure diagnosis [5].

The HP tissue can undergo secondary changes and complications such as acute pancreatitis, pseudo-cyst formation, development of mucinous cysts, and pancreatic intra-epithelial neoplasia [7, 19, 24–26]. Rare cases of ectopic pancreatic tissue complicated by adenocarcinoma or even endocrine tumors have been described. In symptomatic lesions, if a preoperative diagnosis can be secured, minimally invasive techniques should be the treatment of choice, including endoscopic resection and laparoscopic surgery [2, 13, 18]. The case study was thought to be difficult to resect completely by endoscopy and showed invagination; therefore, an open gastrectomy was performed.

## Conclusions

HP can show vertical progression and gastroduodenal invagination. When experimenting with such a tumor, consideration should be given to HP always being included as a part of differential diagnoses. When the diagnosis is difficult, the histological examination, in particular, immuno-histochemical analysis, for example MUC, can reveal pancreatic ectopia, which is a helpful diagnostic method.

## Abbreviations

CA19-9: Carbohydrate antigen 19-9
CEA: Carcinoembryonic antigen
CT: Computed tomography
EUS-FNA: Endoscopic ultrasound-guided fine-needle aspiration
GIST: Gastrointestinal stromal tumor
HP: Heterotopic pancreas
MRI: Magnetic resonance imaging
MUC: Membrane and cytoplasm
NET: Neuroendocrine tumor
SCN: Serous cystic neoplasm
SM: Submucosal
Span-1: S-pancreas-1 antigen
US: Ultrasonography
WI: Weighted image
